# An exploratory analysis of differences in serum protein expression by sex in patients with systemic sclerosis associated interstitial lung disease

**DOI:** 10.1186/s12890-024-03474-z

**Published:** 2025-01-13

**Authors:** Giuliana Cerro-Chiang, Matthew Ayres, Alejandro Rivas, Sarah J. Parker, Mitra Mastali, Peter Chen, Jennifer E. Van Eyk, Paul J. Wolters, Francesco Boin, Tanzira Zaman

**Affiliations:** 1 Section of Pulmonary, Critical Care, and Sleep Medicine, Department of Medicine- Yale School of Medicine, Lippard Laboratory of Clinical Investigation (LLCI), 15 York Street, Rm 101 E, New Haven, CT 06510 USA; 2 Advanced Clinical Biosystems Institute Biomedical Sciences Cedars, Sinai Medical Center, Los Angeles, CA USA; 3 Division of Pulmonary and Critical Care Medicine Cedars Sinai Medical Center, Los Angeles, CA USA; 4 Division of Pulmonary and Critical Care Medicine, University of California San Francisco, San Francisco, CA USA; 5 Division of Rheumatology, Kao Autoimmunity Institute, Cedars Sinai Medical Center, Los Angeles, CA USA

**Keywords:** Systemic sclerosis, Interstitial lung disease, Biomarkers, Sex, Disease progression

## Abstract

**Background:**

Systemic sclerosis (SSc) is a rare connective tissue disease, frequently affecting the skin, lungs, and pulmonary vasculature. Approximately 30–50% of SSc patients develop interstitial lung disease (SSc-ILD), with 30–35% of related deaths attributed to it. Even though men are less likely to develop systemic sclerosis, they have a higher incidence of SSc-ILD than women, and they tend to develop it at a younger age with a higher mortality rate. Sex differences in protein expression in the blood of patients with SSc-ILD have not been reported to date. We aimed to identify sex differences in serum protein expression between men and women with SSc-ILD.

**Methods:**

Serum specimens of patients with SSc-ILD underwent dual mass spectrometry (LC-MS/MS) analysis. The association between protein biomarkers and sex was assessed through logistic regression. Time to event analysis was performed to determine any differences in the time to FVC decline of > 5% and the proportion of subjects who experienced FVC decline of > 5% by sex over the total period of observation. The association between biomarkers and sex was assessed through logistic regression. For proteins that were dichotomized, chi-squared testing was used. Multivariable regression models adjusting for meaningful clinical variables were also performed.

**Results:**

The cohort consisted of 211 subjects, 162 women and 47 men with a median follow-up of 3.52 years. No significant sex differences were found in the time to FVC decline of > 5% or > 10%. Among the 704 proteins identified, forty differed significantly between sexes. After adjusting for multiple testing, Autotaxin remained significantly higher in women. Autotaxin, known to activate lysophosphatidic acid and promote fibrosis, suggests a potential role in modulating fibrotic processes in SSc-ILD.

**Conclusions:**

This study is the first to report sex-specific serum protein differences in patients with SSc-ILD, with Autotaxin remaining significantly different after adjusting for multiple testing. These proteins could influence disease progression and treatment response and underscore the importance of personalized therapeutic strategies and further research into sex-related molecular pathways in SSc-ILD.

**Clinical trial number:**

Not applicable.

**Supplementary Information:**

The online version contains supplementary material available at 10.1186/s12890-024-03474-z.

## Background

Systemic sclerosis (scleroderma or SSc) is a rare autoimmune disease characterized by fibroblast proliferation resulting in vasculopathy, cellular inflammation, and fibrosis. The main organs affected by SSc are the dermis, pulmonary parenchyma and vasculature [1]. Prior cohort studies have estimated the prevalence of SSc-related interstitial lung disease (SSc-ILD) at between 30 and 50% [2, 3] with 30–35% of all scleroderma-related deaths attributed to it [4]. The addition of high-resolution computed tomography (HRCT) in addition to pulmonary function tests has allowed for an earlier diagnosis [5]. Systemic sclerosis is more prevalent in women with a female: male ratio ranging from 5:1 to 12:1 [6]. Even though men are less likely to develop systemic sclerosis, they have a higher incidence of SSc-ILD than women, and they tend to develop it at a younger age [7]. A prior cohort has also identified Asian race as a factor associated with development of impaired diffusing capacity in patients with systemic sclerosis [8].

The trajectory of SSc-ILD also varies by sex. An analysis from the EUSTAR database revealed that at 12 and 24 months, women experienced disease progression more frequently than men. However, men had a higher mortality rate than women at 24 months (30.9% vs. 20.4%) with male gender being an independent predictor of mortality [OR 1.95 (CI 1.66–2.28] [7]. In contrast, a post hoc analysis from the scleroderma lung studies (SLS) I and II, revealed a higher rate of FVC decline at 3–12 months in men compared to women in the SLS I study, although this was not statistically significant. SLS II reported a faster course of FVC decline in men treated with either cyclophosphamide or mycophenolate. Additionally, a greater proportion of men experienced a decline in FVC of over 10% in both studies combined regardless of the treatment arm to which they were assigned [9].

Even though the differences in disease progression and severity differ between men and women, it remains unknown why these differences exist. Differences in protein expression in bronchoalveolar lavages have been described by Volkmann and colleagues, who reported a predominance of pro-fibrotic mediators in men versus a predominance of pro-inflammatory mediators in women [9]. Previously, biomarkers like KL-6 and IL-18 have been associated with the presence and severity of SSc-ILD [5], however, sex differences in protein expression in blood of patients with SSc-ILD have not been reported to date. Given the systemic nature of this disease, we aimed to determine whether there are sex differences in serum protein expression between men and women with SSc-ILD.

## Methods

### Study design and participants

This was a single center, nested cohort study that included subjects diagnosed with SSc-ILD among patients enrolled in the University of California, San Francisco Scleroderma Center registry from September 2015 to June 2020. In order to enter the registry, patients had to fulfil the 2013 American College of Rheumatology/European League Rheumatism criteria for systemic sclerosis [10]. The diagnosis of ILD was determined by radiographic evidence of fibrotic changes on high resolution computed tomography (HRCT) and a forced vital capacity (FVC) of < 80% or a greater than 10% decline in FVC (L) over a twelve-month period.

Demographic data, clinical data including clinical diagnosis, lung function tests, and laboratory tests were extracted from the patient’s electronic medical record. All patients provided Informed consent to be enrolled in the registry. The Institution Review Board at the University of California San Francisco oversaw the compliance of the record, methods, sample storage and collection. In addition, all methods were carried out in accordance with relevant guidelines and regulations.

### Specimen processing and liquid chromatography with dual mass spectrometry (LC-MS/MS) analysis

At the time of the clinical visits, patients provided peripheral blood samples for the registry. The serum was isolated, aliquoted, and stored at -80^o^C. These biobanked serum specimens were used in the proteomics section of the study. To increase yield, the samples were depleted of the 14 most abundant proteins by using the High Select Top 14 Abundant Protein Depletion Camel Antibody Resin (Thermo Fisher Scientific) to enhance the detection and identification of the less abundant proteins and analyzed as outlined by McArdle et al. [11]. After the depletion, the samples underwent digestion with serum trypsin and desalting in preparation to tandem liquid chromatography-mass spectrometry analysis. The proteomics analysis was previously described in our prior work [12]. Data-independent acquisition (DIA) analysis was performed on an Orbitrap Exploris 480 (Thermo Fischer Scientific) mass spectrometer interfaced with a microflow-nanospray electrospray ionization source (Newomics, IS-T01) coupled to an Ultimate 3000 ultra-high-pressure chromatography system. Peptides were loaded at 4 µg, based on average bicinchoninic acid assay (BCA) results, and separated on a gradient of 7–10% B organic phase for 2 min, 10–32% B for 53 min, 32–70% for 1 min, 70–70% for 2.5 min, and then decreased from 70% B to 1% B over 1 min, on a C18 column (15 cm length, 300 μm ID, 100 Å pore size, Phenomenex) for 60 min with a constant flow rate of 9.5 µL/min. We used a mass range of 400–1000 and automated gain control (AGC) target value for fragment spectra of 300%. The peptide ions underwent fragmentation at a normalized collision energy of 30%. The fragmented ions were detected through 50 non-overlapping data independent acquisition precursor windows of 12 Da in size. The MS2 resolution was set to 15,000 with a 22 ms ion transmission time. All the data was acquired in profile mode using positive polarity. In order to mitigate systematic error, the protein estimates were normalized across batches.

### Statistical analysis

Baseline demographics were described using means, standard deviations, medians, interquartile ranges, and proportions. Time to event analysis was performed to determine any differences in the time to FVC decline of > 5% and the proportion of subjects who experienced FVC decline of > 5% by sex over the total period of observation. The threshold of 5% was chosen based on prior studies in idiopathic pulmonary fibrosis and SSc-ILD in which a decline of FVC of over 5% was associated with higher mortality [13–16], and an analysis from the two largest SSc-ILD clinical trials, which showed that a minimally clinically important difference correlated with an FVC decline of 3% [17]. Similar analyses were also performed to determine differences in time to FVC decline of > 10% between the sexes. Multivariable regression models to assess for differences in FVC by sex were also performed, adjusting for clinical variables of race, smoking status, systemic sclerosis subtype, anti-Scl70 and ACA antibody status, baseline FVC (L), and baseline DLCO (cc/sec/mmHg).

For proteins with quantifiable levels in over 50% of the samples, samples with values below the lower level of detection were imputed as one less than the lowest detected value. The protein values were log_10_ transformed for normalization, which was confirmed visually with histograms. When protein levels were detected in less than 50% of the samples, the values were transformed to a dichotomous variable to indicate detected versus not detected. The false discovery rate was calculated using the Benjamini Hochberg method, and P values were adjusted to account for multiple testing. Associations between continuously modelled, log-transformed biomarkers and sex were assessed through logistic regression. For proteins that were dichotomized, chi-squared testing was employed. StataSE 17 software was used for statistical analysis.

## Results

There was a total of 211 subjects, 162 women and 47 men with similar baseline characteristics, Table 1. The median follow-up time for the cohort was 3.52 years [IQR 1.55–6.38], with 3.66 years [IQR 2.13–8.06] for men and 3.33 years [IQR 1.46–6.38] for women. During the observation period, 118 patients experienced a FVC decline of at least 5%. The median time to FVC decline was 2.14 years [IQR 0.99–3.66]. There was no significant difference in time to decline between the sexes (2.48 and 2.09 years in men and women, respectively).

**Table 1:** Demographic characteristics of patient with ILD in the UCSF scleroderma cohort

	Overall	Men (47)	Women (162)	*P* value
Hispanic, n (%)	46 (22)	8 (17)	38 (23)	0.12
Race, n (%)				0.30
Non-Hispanic White	103	25 (53)	78 (48)	
Hispanic	43	8 (17)	35 (22)	
Asian	33	6 (12)	27 (16)	
Black/African American	22	7 (15)	15 (9)	
Pacific Islander/Hawaiian	2	0	2 (1)	
Other	6	1 (2)	5 (3)	
Age at enrollment (SD)	54.65 (12.8)	50.9 (11.6)	51.8 (13.1)	0.65
Anti Scl-70 antibody, n (%)	81 (38)	20 (43)	61 (38)	0.54
Anticentromere antibody, n (%)	10 (5)	2 (4)	8 (5)	0.98
Smoking status, n (%)				0.15
Never	136 (64)	25 (53)	111 (68)	
Current	4 (2)	1 (2)	3 (2)	
Former	71 (34)	21 (45)	48 (30)	
BMI, kg/m^2^, mean (SD)	23.59 (4.8)	24.6 (4.5)	23.3 (4.8)	0.11
Diffuse SSc, n (%)	86 (41)	22 (47)	64 (40)	0.37
Use of Immunosuppression, n (%)	179 (85)	43 (91)	135 (83)	0.16
Presence of pulmonary hypertension, n (%)	51 (24)	14 (30)	37 (22)	0.564
Worst Modified Rodnan skin score [IQR]	3 [3–9]	5 [3–11]	3 [3–8]	0.19
Time from first Raynaud’s symptom onset to ILD diagnosis, years, median [IQR]	4.4 [1.2–10]	2.9 [1.4–5.7]	4.7[1-11.9]	0.17
Time from first non-Raynaud’s symptom onset to ILD diagnosis, years, median [IQR]	2.8 [0.7–6.1]	2.3 [0.7-5]	3 [0.7–6.3]	0.27
Initial FCV, % predicted, mean (SD)	71.3 (19.9)	66.3 (21.1)	72.8 (19.4)	0.06
Initial DLCO, corrected for hemoglobin, % predicted, mean (SD)	55.5 (22.3)	53.2 (24.3)	56.2 (21.7)	0.43

**Fig. 1:**
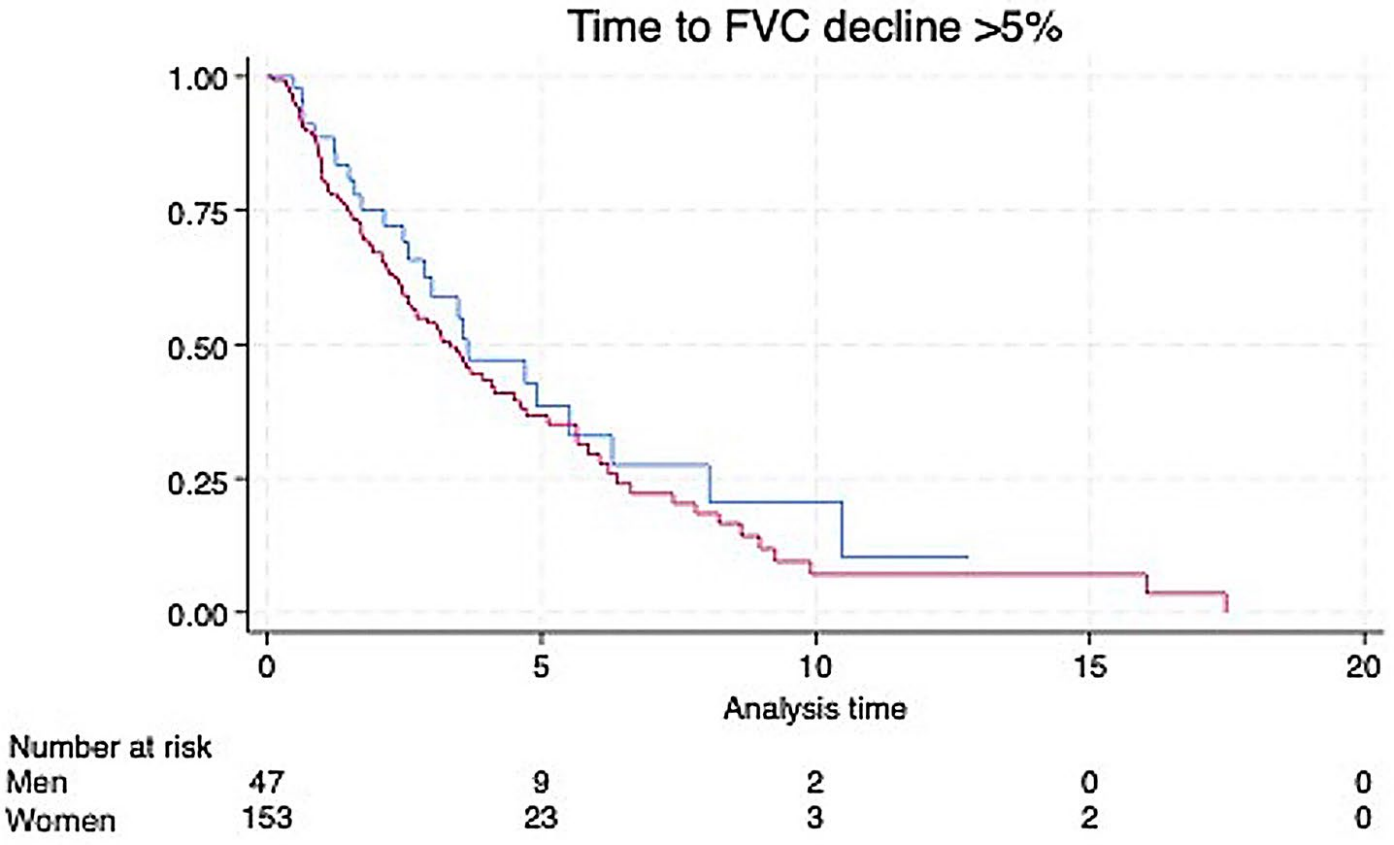
Time to FVC decline of > 5% by sex

There was no significant difference between the percentage of men and women who experienced a decline of over 5% in FVC during the observation period, 52% and 61%, respectively (*p* = 0.38). In a multivariable model adjusting for age, race, smoking status, and Scl-70 positivity, sex was not independently associated with FVC decline. A Kaplan-Meier curve describing the proportion of patients without FVC decline of > 5% by sex is shown in Fig. 1.

**Fig. 2:**
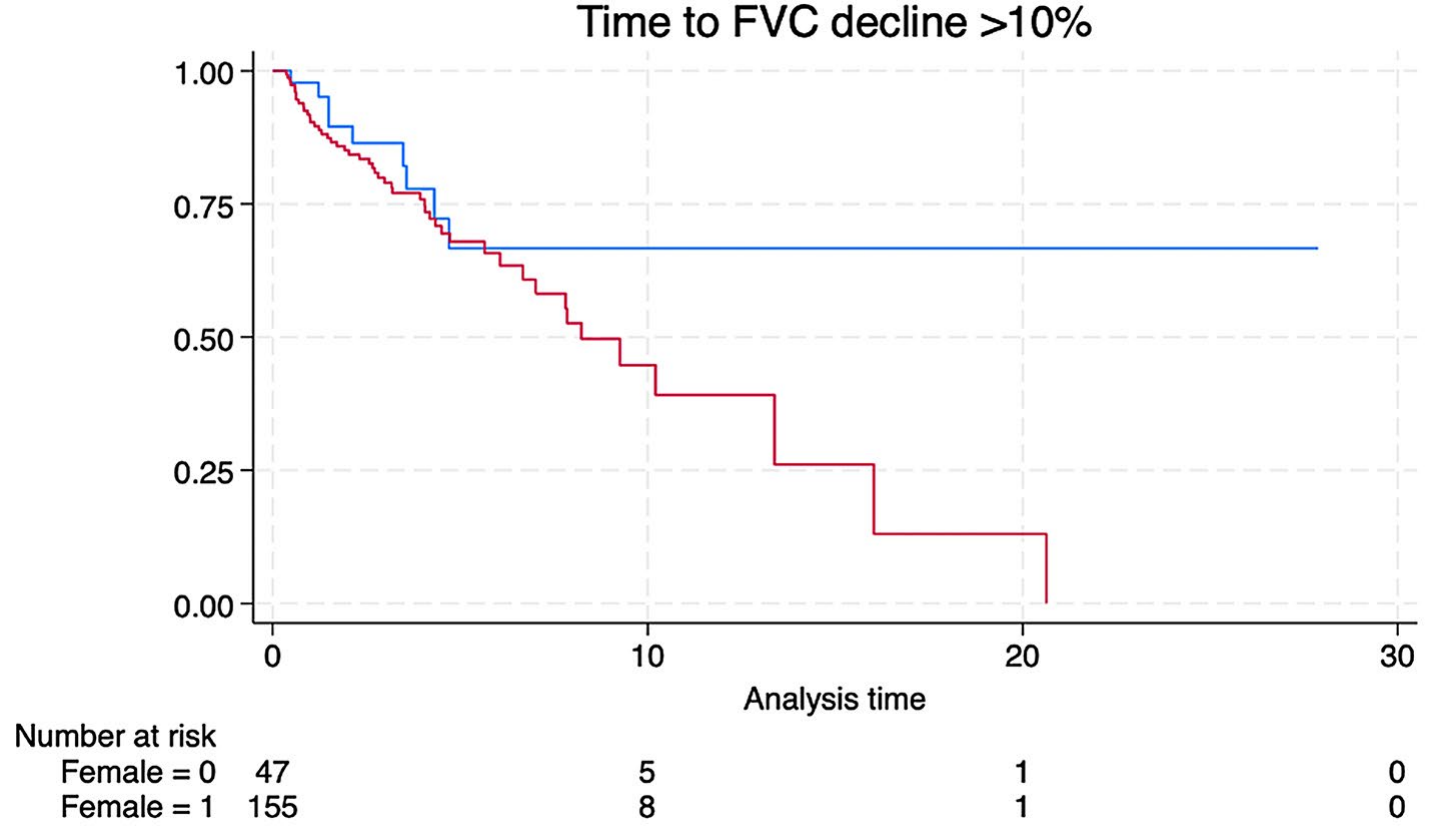
Time to FVC decline of > 10% by sex

Additionally, 58 patients experienced a FVC decline of at least 10%. The median time to FVC decline by 10% was 2.57 years [IQR 1–4.5]. There was no significant difference in median time to decline between the sexes (2.13 and 2.66) years in men and women, respectively. There was no significant difference between the percentage of men and women who experienced a decline of over 10% in FVC during the observation period, 19% and 31%, respectively (*p* = 0.16) with a tendency for women to experience decline. A Kaplan-Meier curve describing the proportion of patients without FVC decline of > 10% by sex is shown in Fig. 2.

### Serum protein differences by sex

Seventy-two patients in our cohort had blood samples available for proteomic analysis.

A total of 794 proteins were identified, of which 652 proteins had quantified values for all patients and 142 proteins in which less than 50% of the sample’s values were below the level of detection. The mean values of forty proteins were significantly different between men and women, and after adjusting for multiple testing, the Ectonucleotide pyrophosphatase/phosphodiesterase family member 2, also known as autotoxin, remained significantly higher in women. Fold differences in mean protein values in women relative to men are presented in Table 2.

**Table 2:** Relative serum protein levels in differentially expressed proteins among men and women with SSc-ILD

Protein full name	Protein symbol	Fold difference in mean protein value in women relative to men	Two-sided *p* value
Azurocidin	AZU1	0.39	0.008
Plastin 1	PLS1	0.45	0.011
Bisphosphoglycerate mutase	BPGM	0.48	0.024
Heat shock protein family A (Hsp70) member 8	HSPA8	0.48	0.026
Phosphogluconate Dehydrogenase	PGD	0.49	0.038
Histone H4	HIST4H4	0.51	0.018
Cathepsin G	CTSG	0.53	0.015
Coronin-1 A	CORO1A	0.57	0.023
Desmocollin-3	DSC3	0.59	0.049
Immunoglobulin Heavy Variable 2-70D	IGHV2-70D	0.60	0.047
Brain abundant membrane attached signal protein 1	BASP1	0.60	0.007
Transaldolase 1	TALDO1	0.61	0.045
Insulin like growth factor binding protein 7	IGFBP7	0.61	0.009
Immunoglobulin heavy variable 1–8	IGHV1-8	0.61	0.025
Leukotriene A4 Hydrolase	LTA4H	0.62	0.008
Phosphatidylethanolamine Binding Protein 4	PEBP4	0.67	0.044
Protein C receptor	PROCR	0.70	0.015
Lipocalin 2	LCN2	0.74	0.049
TIMP metallopeptidase inhibitor 1	TIMP1	0.74	0.039
Protein Tyrosine Phosphatase Receptor Type G	PTPRG	0.80	0.038
Insulin-like growth factor 2 receptor	IGF2R	0.81	0.045
selenoprotein P	SELENOP	0.88	0.039
Complement 5	C5	0.95	0.043
Fibronectin 1	FN1	1.13	0.047
apolipoprotein E	APOE	1.14	0.032
Hepatocyte growth factor activator	HGFAC	1.15	0.020
Insulin-like growth factor (IGF) binding protein-3	IGFBP3	1.20	0.035
Insulin-like growth factor 2	IGF2	1.21	0.028
Protein tyrosine phosphatase receptor type J	PTPRJ	1.25	0.042
Lysosomal associated membrane protein 1	LAMP1	1.25	0.026
Angiopoietin-like 3	ANGPTL3	1.31	0.014
Immunoglobulin Heavy Variable 2–5	IGHV2-5	1.41	0.049
Carnosine Dipeptidase 1	CNDP1	1.42	0.008
Contactin 1	CNTN1	1.46	0.010
Serpin Family A Member 11	SERPINA11	1.57	0.001
Fibrinogen-Like Protein 2	FGL2	1.59	0.035
Ectonucleotide Pyrophosphatase/ Phosphodiesterase 2	ENPP2	1.75	0.000005
immunoglobulin heavy variable 4-38-2	IGHV4-38-2	2.00	0.037
Immunoglobulin Heavy Variable 1-69D	IGHV1-69D	2.07	0.027
DEAD-Box Helicase 46	DDX46	2.98	0.009

## Discussion

Ours is the first study to investigate sex-specific differences in serum protein expression among patients with SSc-ILD. We identified a total of 40 proteins that were significantly different between men and women in patients with SSc-ILD. None of these proteins have been previously associated with sex-related hormones. Additionally, after adjusting for multiple testing, only levels of the protein Ectonucleotide Pyrophosphatase/ Phosphodiesterase 2, also known as autotaxin, remained significantly more elevated in women. Autotaxin is a protein that activates lysophosphatidic acid, which promotes wound healing and collagen deposition. Increased levels of autotaxin have been found in patients with idiopathic pulmonary fibrosis (IPF) and studies in mice have shown that inhibition of ATX lead to reduced lung fibrosis [18, 19]. These findings suggest a potential role for autotaxin in modulating fibrotic processes in SSc-ILD, like its known associations in IPF and other fibrotic disorders. Some of the proteins that did not remain significant after adjusting for multiple testing may still have significance in the pathophysiology of SSc-ILD. For example, Insulin- like growth factor 7 has been associated with type IV collagen binding [5], fibronectin is known to be secreted by fibroblasts, a key cell in the pathogenesis of fibrosis, and acts as a growth factor for them [20] and the role of fibroleukin in lymphocyte function at mucosal sites is currently being studied [21].

Clinically, we did not observe difference in the time to disease progression between men and women in our cohort, whether defined by a FVC decline of over 5% or over 10%. This may be due to the high percentage of patients of both sexes receiving immunosuppression in our cohort, which might have narrowed the differences in disease trajectory. Notably, the EUSTAR cohort suggested that immunosuppression was the strongest protective factor for progression in women whereas in men immunosuppression was not associated with progression, suggesting that the effect might be variable according to sex [7]. Similar findings were seen in the post hoc analysis of SLS I and II, in which women had better response to immunosuppression than men [22]. The differences in response to immunosuppression that have been previously reported could be explained by the differences in proinflammatory or profibrotic protein concentrations between men and women.

Strengths of our study include a well phenotyped cohort, longitudinal data on disease progression, and immunosuppression use which were not previously reported in the literature. *Our small sample size relative to the total number of proteins queried was a major limitation.* Thus, we may have lacked the power to detect statistically significant differences of other proteins. Further studies could evaluate the relationship between these differentially-expressed proteins, including those that did not remain significant after correction for multiple testing and sex, as well as progression of disease.

In conclusion, our study contributes to the data on sex differences in protein expression in SSc-ILD. These differences may provide an opportunity to use biomarkers to personalize immunosuppression or antifibrotic therapy. Overall, these findings should be considered exploratory and further research efforts should focus on evaluation of differential pathways that may define sexual dimorphism in SSc-ILD, particularly in treatment naïve patients.

## Electronic supplementary material

Below is the link to the electronic supplementary material.

Supplementary Material 1

## Data Availability

All data generated or analyzed during this study are included in this published article (and its Supplementary Information files).
